# Functional Characterization of a Single Nucleotide Polymorphism in the 3' Untranslated Region of Sheep DLX3 Gene

**DOI:** 10.1371/journal.pone.0137135

**Published:** 2015-09-02

**Authors:** Enguang Rong, Zhiwei Zhang, Shupei Qiao, Hua Yang, Xiaohong Yan, Hui Li, Ning Wang

**Affiliations:** 1 Key Laboratory of Chicken Genetics and Breeding at Ministry of Agriculture, Key Laboratory of Animal Genetics, Breeding and Reproduction at Education Department of Heilongjiang Province, Harbin, 150030, China; 2 College of Animal Science and Technology, Northeast Agricultural University, Harbin, 150030, China; 3 Institute of Animal Husbandry and Veterinary Institute, Xinjiang Academy of Agriculture and Reclamation Science, Shihezi, 832000, China; 4 State Key Laboratory of Agrobiotechnology, China Agricultural University, Beijing, 100193, China; Institut Jacques Monod, FRANCE

## Abstract

The Distal-less 3 (homeobox protein DLX-3), a transcription factor, is critical for the development of hair follicle and hair formation and regeneration. We previously identified and found that four SNPs (c. *118T>C, c. *228T>C, c. *688A>G and c. *1,038_1,039 insC) in 3′ untranslated region (UTR) of sheep DLX3 were in high linkage disequilibrium with each other and significantly associated with wool crimp (*P*<0.05), however, the underlying mechanisms by which these SNPs affect the wool crimp remains unknown. In the present study, we performed association analysis between these four identified SNPs and DLX3 gene expression in sheep skin using quantitative real-time RT-PCR. The results showed that these SNPs were significantly associated with sheep skin DLX3 mRNA expression levels. Then, we constructed DLX3 3′UTR luciferase reporters and validated the association. The reporter assays showed that the three major haplotypes, derived from the four SNPs, had significantly different effects on luciferase reporter activity and the four SNPs also had significantly different allelic effects on the luciferase reporter activity (p < 0.05). Bioinformatics analysis showed that the SNP (c. *1,038_1,039 insC) was located within a potential miR-188 binding site of the 3′UTR of sheep DLX3 mRNA. This SNP may affect miR-188-mediated DLX3 gene expression and result in phenotypic variation. To test the hypothesis, we investigated the effects of miR-188 mimic and inhibitor on the activity of the DLX3 3′UTR luciferase reporter with different SNP alleles. The results showed that in both sheep fetal fibroblasts (SFFs) and human HaCaT cells, miR-188 mimic could significantly decrease the allele D (deletion) luciferase reporter activity (p < 0.05), but miR-188 inhibitor could increased the reporter activitiy. However, neither miR-188 mimc nor inhibitor could influence the allele I (insertion) reporter activity. In addition, transfection of miR-188 mimic dramatically decreased the endogenous expression of DLX3 in SFFs (p < 0.05). Taken together, we demonstrated that DLX3 is a target gene of miR-188 and the SNP (c. *1,038_1,039 insC) is a functional SNP, and affects miR-188-mediated gene regulation of sheep DLX3. Our finding may in part explain allelic difference in gene expression and wool crimp in our tested sheep population.

## Introduction

The Distal-less 3 (homeobox protein DLX-3, DLX3) is a Distal-less homeodomain protein that belongs to the DLX vertebrate family [1]. Homeobox protein DLX-3 is expressed in placenta, skin and structures involving epithelial-mesenchymal interactions, such as ears, teeth, nails, hair follicles [2]. It has been shown that homeobox protein DLX-3 has an essential role in placental, osteogenic and epidermal development [3–5]. Mutations in human homeobox protein DLX-3 have been found to be responsible for the defects in teeth and bone development called the Tricho-Dento-Osseous syndrome [6,7].

Recent studies have shown that DLX3 is widely expressed in the hair shaft, hair matrix and inner root sheath (IRS) and plays a critical role in hair follicle (HF) development and hair formation [8–10]. Homeobox protein DLX-3 controls differentiation of keratinocyte in hair matrix toward the hair shaft and IRS. Selective ablation of homeobox protein DLX-3 in mice causes failure in formation of the hair shaft and IRS, leading to complete alopecia. It has been shown that homeobox protein DLX-3 regulates the expression of transcription factors crucial for hair follicle differentiation, such as homeobox C13 (HOXC13) and GATA binding protein 3 (GATA3) [9]. During hair follicle differentiation homeobox protein DLX-3 is regulated by bone morphogenetic protein (BMP) and WNT signaling pathways [8,9].

MicroRNAs (miRNAs) are small, single-stranded, evolutionarily conserved, noncoding RNA molecules that bind to the 3′ untranslated region (3′UTR) of target mRNAs via imperfect base pairing, resulting in the cleavage of target mRNAs or repression of their translation [11]. MiRNAs are critical post-transcriptional regulators of gene expression and play important roles in cell proliferation, apoptosis, differentiation, tumorigenesis and many other physiological and pathological processes. Recent findings have revealed miRNA involvement in regulating the morphogenesis and development of skin and epithelial appendage [12]. A number of miRNAs have been found to be involved in the development of epidermis and hair follicles, pigmentation, and hair follicle cycles [13]. Of these miRNAs, miR-31 has been identified to target DLX3, fibroblast growth factor 10 (FGF10), keratin 16 (KRT16) and keratin 17 (KRT17) genes, and regulates hair follicle development and hair fibre formation [8].

The ability of miRNAs to bind to target mRNAs is critical for miRNA target gene regulation. Mounting evidence has shown that the polymorphisms in miRNA binding sites can affect the binding efficacy of miRNAs, leading to the alteration of target gene expression and phenotypes [14,15]. In our previous study, we identified and found that four SNPs (c. *118T>C, c. *228T>C, c. *688A>G and c. *1,038_1,039 insC) and their haplotypes in 3′UTR of sheep DLX3 gene were consistently significantly associated with sheep wool crimp [16]. However, it is unclear how these SNPs affect sheep wool crimp. In the present study, we investigated the possible mechanism by which these SNPs affect sheep wool crimp, and our results showed that the SNP (c. *1,038_1,039 insC) resides in the miR-188 binding site of the 3′UTR of sheep DLX3 mRNA, and affects miR-188-mediated gene regulation of sheep DLX3 gene.

## Materials and Methods

### Animals and tissue collection

Procedures involving animals and their care were approved by Animal Care and Use Committee of the Northeast Agricultural University and Xinjiang Academy of Agriculture and Reclamation Science (China). A total of 26 live ewes from super fine wool strain of Chinese Merino (Xinjiang Junken Type), born between 2005 and 2008, were used in the current study. A local anesthetic was used prior to obtaining the skin samples. One square centimeter of body side skin samples were collected, snap-frozen and stored in liquid nitrogen until extraction of RNA. All the tested sheep were under the same feeding and management regimen.

### Reporter Gene Construction and Site-Directed Mutagenesis

Our previous study indicated that sheep DLX3 3′UTR had three major haplotypes (CCGI, TTAD and TCAD) in our tested population. The 3′UTR fragments containing the three different haplotypes were amplified from individual sheep genomic DNA with the known respective haplotypes using the following primers: (forward), 5′-CCGCTCGAGAACCTCTCACGAAGGAACCC-3′; (reverse), 5′-TTGCGGCCGCGAGGGAGGAGGCTGCTTCT-3′. The forward and reverse primers contained *Xho*I and *Not*I restriction sites at their 5′ ends, respectively. The amplified haplotypes were cloned into *Xho*I and *Not*I sites downstream of SV40 promoter-driven *Renilla* luciferase cassette in psiCHECK2 vector (Promega, Madison, WI, USA) to generate 3′UTR haplotype reporters. The three haplotype reporters were designated as psiCHECK2-CCGI, psiCHECK2-TTAD and psiCHECK2-TCAD, respectively.

In order to examine allelic effects for these four SNPs on reporter gene activity, we generated two more haplotype reporters psiCHECK2-TCGI and psiCHECK2-TCAI by use of a One-Tube Point Mutation Kit (TIANDZ, China). The psiCHECK2-CCGI was used as a template to generate the psiCHECK2-TCGI using the following mutagenic primers: (forward), 5′-GAAAGGACATCCCTTCCCTCTTCCCTTGCCCCTTC-3′, (reverse), 5′-GAAGGGGCAAGGGAAGAGGGAAGGGATGTCCTTTC-3′. The psiCHECK2-TCGI was used as a template to generate psiCHECK2-TCAI with the following mutagenic primers: (forward), 5′-TCCCCCACTCTGGTCAAAGAGGAGGGGTGGGT-3′, and (reverse), 5′-ACCCACCCCTCCTCTTTGACCAGAGTGGGGGA-3′. All the reporter constructs were confirmed by sequencing.

### Cell culture and transient transfection

Primary sheep fetal fibroblasts (SFFs) were cultured as previously described [17], and all the four SNPs (c. *118T>C, c. *228T>C, c. *688A>G and c. *1,038_1,039 insC) were heterozygous in our SFFs cell lines in the present study. Briefly, cells were cultured using Dulbecco’s modified Eagle’s medium: Nutrient Mixture F12 (Gibco-BRL) supplemented with 10% fetal bovine serum (FBS, Gibco), at 37°C, 5% CO_2_ until 60–70% confluence in 12-well plates. SFFs were transfected with the individual reporters using FuGENE HD Transfection Reagent (Roche, Mannheim, Germany) according to the manufacturer’s protocols.

HaCaT cell line (Human adult skin keratinocytes) was purchased from China Center for Type Culture Collection and was maintained in Dulbecco’s modified Eagle’s medium (Gibco-BRL) supplemented with heat-inactivated 10% FBS in an atmosphere of 5% CO_2_ at 37°C, until 60–70% confluence in 12-well plate. Lipofectamine 2000 (Invitrogen, Paisley, UK) was used for cotransfection of the DLX3 3′UTR reporters with synthetic miRNA mimic, miRNA inhibitor (anti-miRNA) or miRNA negative controls (Shanghai GenePharma, China) into HaCaT cells and SFFs. The following sequences for miRNA mimics negative control: (sense), 5′-UUCUCCGAACGUGUCACGUTT-3′, (antisense), 5′-ACGUGACACGUUCGGAGAATT-3′; and miRNA inhibitor negative control: 5′-CAGUACUUUUGUGUAGUACAA-3′. Cells were harvested 48 h after transfection for further analyses.

### RNA extraction and real-time RT-PCR

Total RNA from the tissues and cells was isolated using Trizol (Invitrogen, Rockville, MD), and RNA quality was assessed by electrophoresis. Then RNA was DNase-treated and cDNA was synthesized with Oligo(dT)18 primer using the Promega Improm-II reverse transcriptase following the manufacturer’s instructions. The synthesized cDNA was used as a temple for quantitative real-time RT-PCR. Quantitative real-time RT-PCR was carried out using the 7500 real-time PCR System (Applied Biosystem) and Faststart Universal SYBR Green Master (Roche) following the protocol provided by the manufacturer. The housekeeping gene glyceraldehyde-3-phosphate dehydrogenase (GAPDH) was used as an internal control for gene expression analysis. The primer sequences of the studied genes are shown in Table 1. The miRNA expression was determined using miRcute miRNA first-strand cDNA synthesis kit and miRcute miRNA qPCR detection kit (TIANGEN, China) following the manufacturer’s protocol. The following primers were used for miRNA expression analysis: miR-31: 5′-CAGGCAAGATGCTGGCATAGCT-3′; miR-188: 5′-ATCCCTTGCATGGTGGAGGGT-3′. 5S ribosomal RNA (5S) was used as an internal control for miRNA expression analysis. The following primer sequences for 5S: forward, 5′-CTCGTCTGATCTCGGAAGCTAA-3′ and reverse, 5′-CTACAGCACCCGGTATTC-3′. Their specificities were validated by a single peak in their thermal dissociation curve. For each gene or miRNA of interest, all real-time RT-PCR was performed in triplicate. Gene expression differences between samples and controls were calculated using 2^−ΔΔCT^ method, and the relative expression of DLX3 in skin was calculated using 2^−ΔCT^ method.

**Table 1:** Primer pairs used for quantitative Real-Time RT-PCR.

Gene	Genbank Accession No.	Forward/reverse primer sequences (5′-3′)
DLX3	FJ654646	F: CCACCAGTTCAATCTCAACGGGCTT
		R: GGCTTTCGGACCTTCTTGGGCTTC
HOXC13	EU839660	F: TACCAGCACTGGGCTCTCTC
		R: AGCTGCACCTTGGTGTAGGG
GATA3	NM_001252183	F: CCACAAGATGAACGGACAGAACC
		R: TGTGGTTTGACAGTTTGCAC
KRT71	JF309097	F: TCAGATCCAGTCCCACATCA
		R: GTACAGGGCCTCAGCCTCAG
KRT25	NM_001009739	F: GCTCTTCATCAGAGCGTAGA
		R: GAACCTGCATCTCCTCTTTA
KRT27	NM_001075815	F: CAGCAGCAGATTTCTGATGA
		R: CAGTAGTTGCCCTCCGTCTC
LEF1	HM059927	F: AGAGGAAGGTGACTTAGC
		R: CGGGCACTTTATTTGATGTT
GAPDH	NM_001190390	F: CTGACCTGCCGCCTGGAGAAA
		R: GTAGAAGAGTGAGTGTCGCTGTT

### Bioinformatics analysis

The DLX3 3′UTR sequence was analyzed for putative miRNA binding sites and other important cis-acting elements using the following softwares: Microinspector (http://bioinfo.uni-plovdiv.bg/microinspector/); miRBase (http://www.mirbase.org/index.shtml); TargetScan (http://www.targetscan.org/); and UTRdb (http://utrdb.ba.itb.cnr.it/).

### Luciferase reporter gene assay

SFFs and HaCaT cells were seeded in 12-well plates 24 h before transfection. The individual haplotype reporter constructs were transfected into SFFs and HaCaT cells, respectively. For contrasfection, the individual haplotype reporter constructs (1.6 μg), together with 80 nM miRNA mimic, miRNA inhibitor or negative control, were transfected into SFFs and HaCaT cells, respectively. At 48 h after transfection or contransfection, the relative luciferase activities were determined using Dual-Glo Luciferase Assay System (Promega). Relative reporter activity was obtained by normalization to *Firefly* luciferase activity (the ratio of *Renilla* luciferase to *Firefly* luciferase). All reporter gene assays were performed in triplicate. Data are representative of at least two independent experiments.

### Statistical analysis

According to the characteristic of the tested Super fine wool strain population, models used for analyses were assumed to be: *Y* = *μ* + *G* + *A* + *G*×*A* + *e*. Where *Y* is the observed value; *μ* is the population mean; genotype (*G*) and age (*A*) were the fixed effects; *G* × *A* was the interaction effects of *G* by *A*; *e* was the random error. Data were subjected to the GLM procedures of JMP5.1 (SAS Institute, Cary, NC, USA) to examine the correlation between genotypes and gene expression levels and to evaluate the least square means. Student’s *t*-test was used to determine the significant differences between groups. P values < 0.05 were considered statistically significant, and p values < 0.01 were considered highly significant.

## Results

### Association of 3′UTR SNPs with DLX3 mRNA expression levels

Previously, we demonstrated that the four identified SNPs (c. *118T>C, c. *228T>C, c. *688A>G and c. *1,038_1,039 insC, designated as SNPs 1 to 4, respectively) in 3′UTR of DLX3 were essentially linked together and were highly associated with wool crimp in the tested Chinese Merino populations [16]. To understand how these SNPs affect sheep wool crimp, we first performed the association analyses of these SNPs with the skin DLX3 mRNA expression. A total of 26 live ewes from super fine wool strain of Chinese Merino (Xinjiang Junken Type) were used in this study. DLX3 genotyping showed that the four SNPs were coincidentally in complete LD, i.e, only two haplotypes (TTAD and CCGI) existed in these tested 26 individuals. Association analysis showed that all these four SNPs (equivalent to one SNP) had significant impact on the DLX3 mRNA expression (p = 0.0160), e.g., for SNP4 (c. *1,038_1,039 insC), its two alleles (D and I) had significantly different effects on DLX3 mRNA expression levels, the sheep with genotypes DD and DI had much lower DLX3 mRNA expression levels than did sheep with genotype II (Table 2). Surprisingly, we observed that the expression of DI genotype is half the expression of the DD genotype and not intermediate between DD and II genotypes. The bizarre results may be caused by the small sample size and high variations in DLX3 expression in the tested samples. In addition, there were no significantly different effects on DLX3 mRNA expression between the two reciprocal haplotypes (TTAD and CCGI) (p = 0.2629). Due to complete linkage of these four SNPs, these data suggest that some of these SNPs are functional and affect DLX3 gene expression.

**Table 2:** Multivariate associations of factors with skin DLX3 mRNA expression levels.

Factors	Level of factors (n^2^)	Relative expression^3^	p value
Genotype^1^ (c. *1,038_1,039 insC)	DD(8)	0.01729286±0.00838556^b^	p = 0.0160*
	DI(13)	0.00750117±0.00568264^b^	
	II(5)	0.05062146±0.01182850^a^	
Age	1(8)	0.022233±0.014334	p = 0.3822
	2(10)	0.013147±0.008257	
	3(5)	0.015200±0.011561	
	4(3)	0.012643±0.008911	

^1^ The four studied SNPs (c. *118T>C, c. *228T>C and c. *688A>G and c. *1,038_1,039 insC) were in complete LD in the 26 tested sheep, and they all were significantly associated with skin DLX3 mRNA expression levels. Table 2 just shows the association analysis results of SNP c. *1,038_1,039 insC, which is good surrogates for other SNPs. Three genotypes for c. *1,038_1,039 insC were detected and defined as DD (deletion), DI, and II (insertion), respectively.

^2^ Numbers shown in parentheses are the number of individuals with the specified genotype or age.

^3^ The relative skin DLX3 mRNA expression was calculated using 2^-ΔCt^ method. The values are represented as least square means ± standard errors.

^a, b^ Means within a row with no common superscript are different (p < 0.05).

* indicates p < 0.05.

### Effects of the haplotypes of DLX3 3′UTR on reporter gene activity

**Fig 1:**
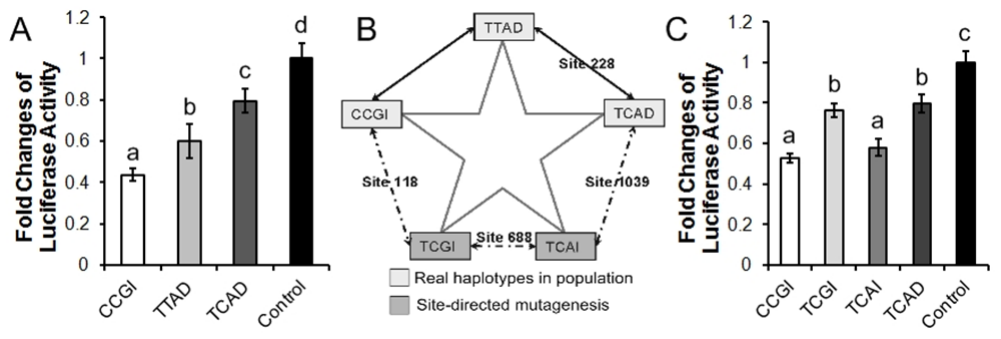
Effects of three major haplotypes and alleles of the four SNPs on DLX3 3′UTR reporter gene activity. (**A**) Relative luciferase activities of the three major haplotypes of sheep DLX3 3′UTR. The reporter activity was expressed as relative luciferase activity (Rluc/Fluc ratio). The psiCHECK2 empty vector was used as a control. Different letters (a, b, c and d) above columns indicate significant difference between groups (p < 0.05). (**B**) Strategy applied to construct novel haplotypes. Three major haplotypes (CCGI, TTAD and TCAD) were present in our tested population. In order to investigate the allelic effects of each of these four SNPs, two novel haplotypes (TCGI and TCAI) were constructed by use of a One-Tube Point Mutation Kit (TIANDZ, China). The allelic effects of SNPs 1 to 4 on reporter gene activity were measured by comparing between CCGI and TCGI for SNP1, between TTAD type and TCAD for SNP2, between TCGI and TCAI for SNP3, and between TCAI and TCAD for SNP4, respectively. (**C**) Relative luciferase activity of the haplotypes of sheep DLX3 3′UTR. The psiCHECK2 empty vector was used as a control. Different letters (a, b and c) above columns indicate significant difference between groups (p < 0.05). All reporter assays were performed in SFFs. Data are representative of at least three independent experiments (error bars, S.D.).

To functionally validate the association of the identified SNPs with the sheep skin DLX3 expression, we cloned the 3′UTRs of the three major haplotypes (CCGI, TTAD and TCAD) based on these four SNPs, and generated their respective 3´UTR haplotype reporters. Reporter assays showed that reporter activities were significantly different among these three major haplotypes (p < 0.05) (Fig 1A). Of these three major haplotypes, CCGI haplotype reporter had the lowest reporter activity, and TCAD haplotype reporter had the highest reporter activity in SFFs. Consistent with the association results,these reporter assay results also suggest that some of these SNPs are functional and affect DLX3 gene expression. Then we generated two more novel haplotype reporters (psiCHECK2-TCGI and psiCHECK2-TCAI) using site-directed mutagenesis (Fig 1B) and detected the allelic effects of the four SNPs on luciferase reporter activities by comparing between psiCHECK2-CCGI and psiCHECK2-TCGI for SNP1, between psiCHECK2-TTAD and psiCHECK2-TCAD for SNP2, between psiCHECK2-TCGI and psiCHECK2-TCAI for SNP3, between psiCHECK2-TCAI and psiCHECK2-TCAD for SNP4, respectively. Surprisingly, these four SNPs had significantly different allelic effects on the luciferase reporter activity (Fig 1A and 1C) (p < 0.05), suggesting all of these four SNPs may additively or synergistically affect reporter gene expression posttranscritionally.

### Bioinformatics analysis of 3′UTR of sheep DLX3 gene

The 3′UTR of sheep DLX3 gene is approximately 1.5 kb long, which is more than 1.5 times longer than that of human DLX3 (1.02 kb) [18] and accounts for more than half of the full-length DLX3 mRNA (2.5 kb), suggesting 3′UTR may play an important role in the regulation of DLX3 expression. To understand the molecular mechanism by which these SNPs affect DLX3 gene expression, we first performed bioinformatics analysis using UTR databases. The results showed that there were a number of motifs in the 3′UTR of sheep DLX3, such as SXL binding site (SXL_BS, c. *1,199–1,209), Musashi binding element (MBE, c. *1,189–1,193), Polyadenylation Signal (PAS, c. *1,485–1,502) and 3 reiterations of the signature AU rich elements (ARE) pentamer sequence AUUUA (c. *164–168, c. *576–580 and c. *1,253–1,257). Unfortunately, these four SNPs were not located within these known motifs.

However, using the human, mouse, cow and sheep miRNA databases, we found these four individual SNPs were located within or near a number of potential miRNA binding sites (S1 Table). This prompted us to hypothesize that some of these SNPs may affect the binding of miRNA to 3′UTR of DLX3 mRNA, ultimately resulting in the alteration of DLX3 gene expression. Among the list of predicted miRNAs (S1 Table), only miR-188 is well known for its expression and involvement in hair follicle [19,20]. MiR-3957-5p was the only one miRNA reported in the sheep miRNA database. SNP3 and SNP4 were predicted to be located within the putative binding sites of miR-3957-5p and miR-188, respectively, in the 3′UTR of sheep DLX3 mRNA. Therefore, in the subsequent study, we tested whether these two SNPs (SNP3 and SNP4) affect miRNA regulation of sheep DLX3 by miR-3957-5p and miR-188.

### Effects of SNPs on miRNA regulation of sheep DLX3 gene

**Fig 2:**
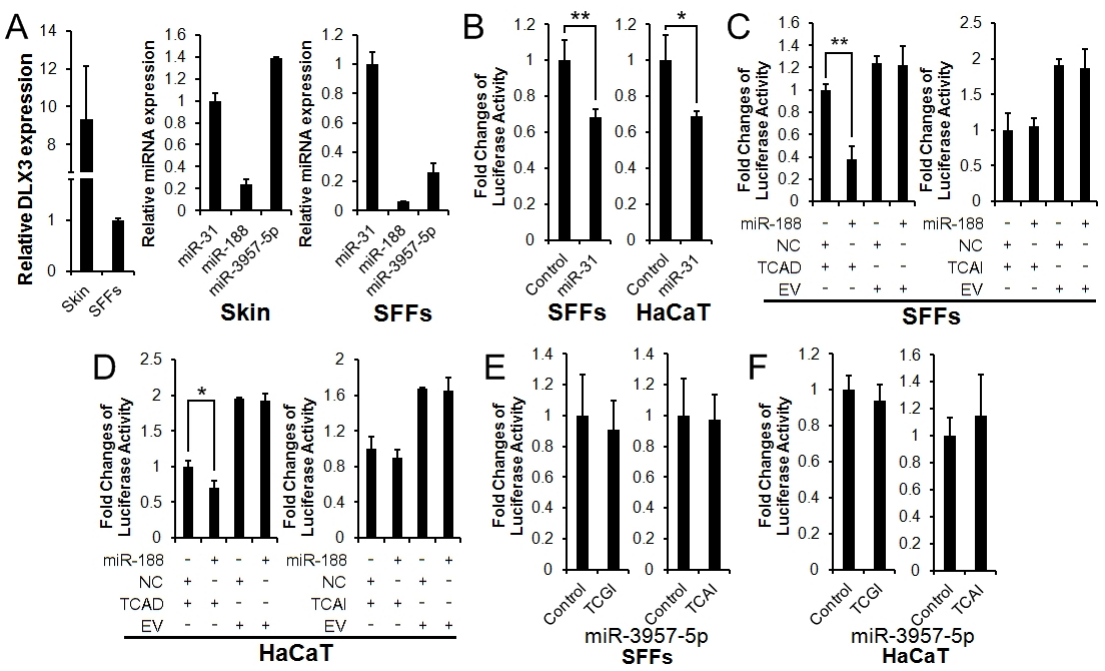
Effects of miR-188 on DLX3 3'UTR Luciferase Reporter activity. (**A**) Expression analysis of DLX3 and miRNAs in sheep skin and SFFs by real-time RT-PCR. The data were normalized against GAPDH (gene expression, left panel) and 5S ribosomal RNA (miRNA expression, middle and right panels), respectively. All PCRs were performed in triplicate; the data are expressed as the mean ± S.D. of three independent experiments. (**B**) Effects of miR-31 on DLX3 3'UTR luciferase reporter activity in SFFs and HaCaT cells, respectively. The graph is a mean of the three haplotypes (CCGI, TTAD or TCAD). About >30% suppression in the reporter activity was observed, compared to the control (cells cotransfected with psiCHECK2 vector and miR-31 mimic). (**C**, **D**) Effects of miR-188 mimic on DLX3 3'UTR reporter activity of psiCHECK2-TCAD and psiCHECK2-TCAI reporters in SFFs and HaCaT cells. MiR-188 mimc reduced the reporter activity of psiCHECK2-TCAD reporter by over 50%, but did not alter the reporter activity of psiCHECK2-TCAI reporter. NC: miRNA mimics negative control. EV: psiCHECK2 empty vector. (**E**, **F**) Effects of miR-3957-5p mimic on the reporter activity of psiCHECK2-TCGI and psiCHECK2-TCAI reporters in SFFs and HaCaT cells. MiR-3957-5p mimic caused no significant changes in the reporter activity of both psiCHECK2-TCGI and psiCHECK2-TCAI reporters. *p < 0.05; **p < 0.01.

We first performed miRNA and gene expression analysis in sheep skin and SFFs, and the results showed that miR-188, miR-3957-5p, miR-31 and DLX3 were to some extent expressed in both sheep skin and SFFs (Fig 2A), implying that it is possible that miR-188 and miR-3957-5p regulate sheep DLX3 gene posttranscriptionally.

To further test whether miR-188 and miR-3957-5p directly regulate DLX3 expression and whether SNP4 and SNP3 affect miRNA regulation of sheep DLX3, we performed luciferase reporter assays in both SFFs and HaCaT cells using miRNA mimics. MiR-31 has been shown to target DLX3 in hair follicle [8] and none of the four studied SNPs are locate within the miR-31 binding site. In the present study, miR-31was selected as a positive control, and miRNA repression of average luciferase activity ≥ 30% was considered a significant effect on gene expression [21]. The miRNA mimics efficacy was assessed by real-time RT-PCR, which revealed a significant increase in the respective miRNA level in the SFFs individually transfected with miRNA mimic of miR-31, miR-188 and miR-3957-5p, compared to the cells transfected with the mimic negative controls (S1A Fig). The reporter gene assay showed that as expected, miR-31 mimic could cause > 30% suppression of the luciferase activities of the 3′UTR luciferase reporters (psiCHECK2-CCGI, psiCHECK2-TTAD or psiCHECK2-TCAD) in both SFFs and HaCaT cells, compared to the negative control (Fig 2B), consistent with the previous report in mice [8].

Similarly, our results showed that, miR-188 mimic could cause > 50% reduction of reporter activity of the allele D reporter (psiCHECK2-TCAD) containing the putative miR-188 binding site in SFFs and HaCaT cells, compared to its mimic negative control (NC). In contrast, miR-188 mimic could not cause significant reduction in reporter activity of allele I reporter (psiCHECK2-TCAI) containing the mutated putative miR-188 binding site, compared to its mimic negative control (Fig 2C and 2D). Taken together, these data suggest miR-188 may regulate sheep DLX3 and SNP4 affects DLX3 gene expression. In contrast, for the two alleles (G and A) of SNP3, miR-3957-5p mimic had no clear effect on the reporter activity of both allele G and A reporters (psiCHECK2-TCGI and psiCHECK2-TCAI) in both SFFs and HaCaT cells, compared to negative control (psiCHECK2, Fig 2E and 2F) suggesting miR-3957-5p does not regulate DLX3 expression.

**Fig 3:**
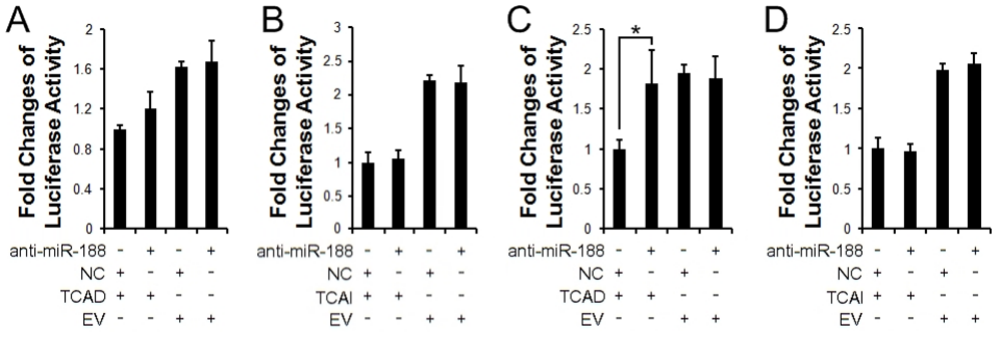
Effect of miR-188 inhibitor on the reporter activity of psiCHECK2-TCAD and psiCHECK2-TCAI reporters. (**A**, **B**) Effect of miR-188 inhibitor on reporter activity of psiCHECK2-TCAD and TCAI in SFFs. MiR-188 inhibitor increased the luciferase activity of psiCHECK2-TCAD (**A**), not psiCHECK2-TCAI **(B)** in SFFs, compared to the negative control. (**C**, **D**) Effect of miR-188 inhibitor on the reporter activity of psiCHECK2-TCAD and psiCHECK2-TCAI reporters in HaCaT cells. MiR-188 inhibitor increased reporter activity of psiCHECK2-TCAD (**C**), not psiCHECK2-TCAI **(D)** in HaCaT cells, compared to the negative control. NC: miRNA inhibitor negative control. EV: psiCHECK2 empty vector. *p < 0.05.

To further validate whether miR-188 targets sheep DLX3 3'UTR and whether SNP4 affects miR-188-mediated regulation of DLX3 gene, we tested the effect of miR-188 on DLX3 3'UTR reporter activity using miRNA synthetic inhibitors, a class of chemically engineered oligonucleotides that prevent endogenous miRNA. First, we checked whether miR-188 inhibitor could reduce endogenous miR-188 expression. The result showed that miR-188 inhibitor (anti-miR-188) indeed could significantly reduce miR-188 expression in SFFs compared to the control (S1B Fig). Then we tested the effect of anti-miR-188 on the reporter activity of two individual haplotype reporters (psiCHECK2-TCA***D*** and psiCHECK2-TCA***I***). As shown in Fig 3A, anti-miR-188 increased reporter activity of psiCHECK2-TCAD (allele D) reporter in SFFs compared to miRNA inhititor negative control. Similarly, in HaCaT cells, anti-miR-188 significantly increased the reporter activity of psiCHECK2-TCA***D*** by 1.8-fold (p < 0.05), compared to its negative control (Fig 3C). However, anti-miR-188 had no clear effect on reporter activity of psiCHECK2-TCA***I*** (allele I) reporter in both SFFs and HaCaT cells (Fig 3B and 3D).

Finally, we investigated the effect of miR-188 mimic on endogenous expression of DLX3 and some other hair follicle–associated genes in SFFs. The real-time RT-PCR expression analysis showed that, similar to miR-31, in agreement with reporter gene findings, miR-188 mimc reduced the endogenous DLX3 gene expression in SFFs, compared to the mimics negative control (S2A Fig), which is in agreement with reporter gene findings. In addition, miR-188 decreased the endogenous expression of hair follicle–associated genes HOXC13, GATA3, keratin 71 (KRT71) and lymphoid enhancer binding factor 1 (LEF1) (S2B–S2E Fig). Taken together, all these results demonstrated that miR-188, not miR-3957-5p, directly targets DLX3, and SNP4 affects miR-188-mediated downregulation of DLX3.

## Discussion

The hair follicle morphogenesis and development is regulated by a complex network of gene functions and signaling pathways. The hair follicle determines hair shaft structure and shape, such as hair length, fineness and crimp [22,23]. Homeobox protein DLX-3 is essential for hair follicle morphogenesis, differentiation and cycling [9]. A recent study showed that DLX3 gene is a direct target gene of the transcription factor Hairless (HR) and HR affects IRS keratin expression via regulation of homeobox protein DLX-3, thereby modulating the formation of IRS of hair follicle [10]. Previously, we found that the four SNPs and their haplotypes in 3′UTR of sheep DLX3 gene were consistently and significantly associated with wool crimp in the tested population [16], however, it is unknown how these SNPs affect sheep wool crimp. In the present study, we demonstrated that the SNP4 (c. *1,038_1,039 insC) affects miR-188-mediated regulation of DLX3, possibly contributing to the wool crimp variation.

In the present study, we demonstrated that miR-188 directly target DLX3 and SNP4 affects miR-188-mediated regulation of DLX3 gene expression. The evidence is as follows: First, here is a putative miR-188 binding site in the 3′UTR of sheep DLX3, and miR-188 and DLX3 were coexpressed in sheep skin and SFFs. Second, the reporter gene assays showed that in SFFs (mesenchymal cells) and HaCaT cells (epidermal cells), miR-188 mimic decreased its allele D reporter (psiCHECK2-TCAD) activity (Fig 2C and 2D), and miR-188 inhibitor increased its allele D reporter activity (Fig 3A and 3C), and neither miR-188 mimic nor miR-188 inhibitor had significant effect on its allele I reporter activity (Figs 2E, 3B and 3D). Finally, miR-188 decreased the endogenous DLX3 mRNA expression in SFFs.

Bioinformatics analysis also showed that there was a putative miR-3957-5p binding site in the 3′UTR of sheep DLX3 gene, and SNP3 was located within this putative miR-3957-5p binding site. We hypothesized that SNP3 might affect miR-3957-5p-mediated regulation of DLX3 gene expression. However, we failed to confirm this hypothesis. MiR-3957-5p mimc did not affect the reporter activity of the allele G and A reporters (psiCHECK2-TCGI and psiCHECK2-TCAI) (Fig 2E) suggesting that DLX3 is not a target of miR-3957-5p, and SNP3 may be linked to a causative mutation or it may function via some unknown mechanisms to affect DLX3 gene expression and the wool traits.

The 3′UTRs of eukaryotic mRNAs play important roles in post-transcriptional regulation of gene expression, including modulation of mRNA transport, mRNA stability, translational efficiency and subcellular localization [24–26]. MiRNAs bind to the 3′UTRs of their target mRNAs, and post-transcriptionally repress gene expression by mRNA degradation and/or translational inhibition. Accumulating evidence show that SNPs within miRNA binding sites affect miRNA-mediated gene regulation, contributing to gene expression and phenotype variation [27–30]. In the present study, the association analysis showed that the four identified SNPs in the 3′UTR of DLX3 were significantly associated with sheep skin DLX3 mRNA expression levels. Consistently, reporter assays showed that these SNPs had significant allelic differences in luciferase reporter activity (Fig 1A and 1C). Taken together, these data indicate that some of these four SNPs are functional and may function additively or synergistically.

In the present study, the association analysis (Table 2) and reporter gene assays (Figs 1–3) suggest that some of these four identified SNPs are functional and affect DLX3 gene expression. However, we just provided evidence that the SNP4 affects DLX3 gene regulation, at least in part, by interfering with miR-188-mediated downregulation of DLX3 gene. How the other three SNPs affect DLX3 gene expression and phenotype remain unknown. The regulatory motifs located in 3′UTRs interact with specific RNA-binding proteins and specific complementary non-coding RNAs including miRNAs, and regulate posttranscriptional gene expression. Their interaction depends on a combination of 3′UTR primary and secondary structure. These four identified SNPs may dependently or independently affect the interaction between 3′UTRs with RNA-binding proteins and non-coding RNAs, and function additively or synergistically. The mechanisms by which these SNPs affect DLX3 gene expression need to be further explored in the future. DLX3 is a downstream target of the WNT and BMP signaling pathways and an essential regulator of the formation of the hair shaft and inner root sheath [9]. Our results showed that sheep DLX3 is a target of miR-188, and the SNP4 in 3′UTR of sheep DLX3 affects miR-188-mediated downregulation of DLX3 (S2A Fig), possibly contributing to the wool trait variation in our tested population. Accumulating evidence show that miRNAs and their mRNA targets form a complex regulatory network. A single miRNA can bind to and regulate many genes as its target, and one gene can be regulated by many different miRNAs [31,32]. Here, we detected the effect of miR-188 on the endogenous expression of DLX3 and other several hair follicle-associated genes. The results showed that, similar to miR-31, miR-188 regulated the expression of these genes (HOXC13, GATA3, KRT71 and LEF1) in SFFs (S2B–S2E Fig). These expression data suggest that miR-188 may be a new important regulator in hair follicle development and hair formation.

In summary, our findings for the first time demonstrate that DLX3 is a target of miR-188, and the SNP c. *1,038_1,039 insC affects miR-188-mediated downreguation of sheep DLX3 gene, possibly contributing to wool phenotypic variation. This SNP may be used as a functional marker for sheep molecular marker-assisted selection.

## Supporting Information

S1 Fig Overexpression and knockdown efficacy of miRNA mimcs and inhibitor in SFFs. (DOC)

S2 Fig MiR-31 and miR-188 downregulate the gene expression of DLX3, HOXC13, GATA3, KRT71 and LEF1 mRNA in SFFs. (DOC)

S1 Table Bioinformatic prediction of SNPs within putative miRNA binding sites of the 3′UTR of sheep DLX3. (DOC)
