# Supplementary material for: Functional Characterization of a Single Nucleotide Polymorphism in the 3' Untranslated Region of Sheep DLX3 Gene
Source: PLoS One. 2015 Sep 2;10(9):e0137135. doi: 10.1371/journal.pone.0137135 (PMC4558038; doi:10.1371/journal.pone.0137135)
Supplement: S1 Fig — (DOC) [file pone.0137135.s001.doc]

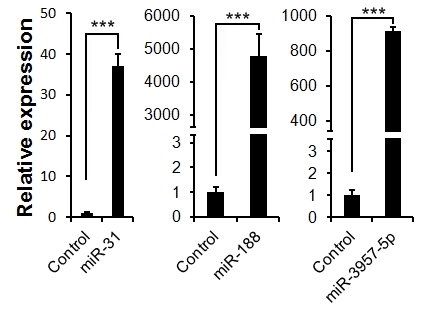

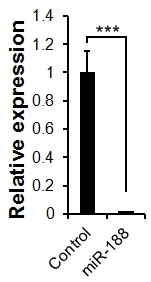


**S1 Fig. Overexpression and knockdown efficacy of miRNA mimcs and inhibitor in SFFs.** (**A**) Overexpression efficacy of miRNA mimcs in SFFs. SFFs were transfected with the miRNA mimics of miR-31, miR-188 and miR-3957-5p, respectively. And miRNA expression was determined 2 days after transfection by quantitative real-time RT-PCR. (**B**) Knockdown efficacy of miR-188 inhibitor. SFFs were transfected with miR-188 inhibitor, and miRNA expression was determined 2 days after transfection by quantitative real-time RT-PCR. All the values are the average of three independent experiments, each measured in duplicate (mean ± S.D.). ***p < 0.001.

A

B
