# Supplementary material for: Functional Characterization of a Single Nucleotide Polymorphism in the 3' Untranslated Region of Sheep DLX3 Gene
Source: PLoS One. 2015 Sep 2;10(9):e0137135. doi: 10.1371/journal.pone.0137135 (PMC4558038; doi:10.1371/journal.pone.0137135)
Supplement: S2 Fig — (DOC) [file pone.0137135.s002.doc]

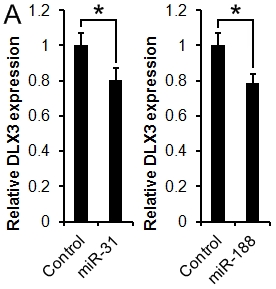

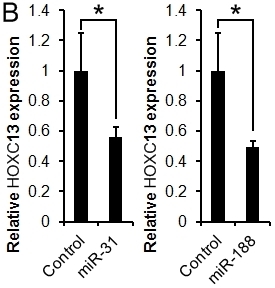

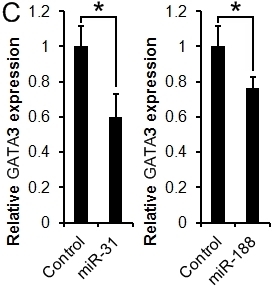


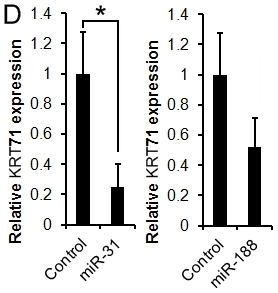

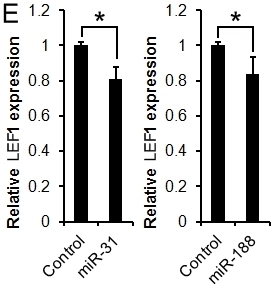


**S2 Fig. MiR-31 and miR-188 downregulate the gene expression of DLX3, HOXC13, GATA3, KRT71 and LEF1 mRNA in SFFs.** (**A**-**E**) Downregulation of DLX3, HOXC13, GATA3, KRT71 and LEF1 mRNA in the SFFs transfected with miR-31 and miR-188 mimics, respectively, compared to the negative control, as determined by real-time RT-PCR. All the values were the average of three independent experiments, each measured in duplicate (mean ± S.D.). *p < 0.05.
