# Supplementary material for: Functional Characterization of a Single Nucleotide Polymorphism in the 3' Untranslated Region of Sheep DLX3 Gene
Source: PLoS One. 2015 Sep 2;10(9):e0137135. doi: 10.1371/journal.pone.0137135 (PMC4558038; doi:10.1371/journal.pone.0137135)
Supplement: S1 Table — (DOC) [file pone.0137135.s003.doc]

S1 Table. Bioinformatic prediction of SNPs within putative miRNA binding sites of the 3′UTR of sheep DLX3

| c. *118T>C | c. *228T>C | c. *688A>G | c. *1,038_1,039 insC |
| --- | --- | --- | --- |
| bta-miR-2406 | bta-miR-2882 | bta-miR-2436-3p | rno-miR-3557-5p |
| bta-miR-2426 | rno-miR-290 | hsa-miR-4638-3p | rno-miR-483 |
| rno-miR-204* | mmu-miR-326* | hsa-miR-3619-3p | bta-miR-188 |
| mmu-miR-326* | hsa-miR-1226* | hsa-miR-4749-3p | mmu-miR-3971 |
| mmu-miR-204* | **bta-miR-1777b** | **oar-miR-3957-5p** | mmu-miR-1188* |
| mmu-miR-678 | **bta-miR-763** | **bta-miR-2328** | mmu-miR-3058 |
| hsa-miR-4739 | **bta-miR-491** |  | hsa-miR-4649-3p |
| hsa-miR-765 | **bta-miR-658** |  | hsa-miR-1236 |
| hsa-miR-939 | **rno-miR-3584-5p** |  | hsa-miR-1909* |
| hsa-miR-149* | **rno-miR-3562** |  | **rno-miR-3581** |
| hsa-miR-4651 | **rno-miR-347** |  | **rno-miR-324-3p** |
| hsa-miR-4271 | **mmu-miR-5120** |  | **rno-miR-128-1*** |
| hsa-miR-1587 | **mmu-miR-491** |  | **rno-miR-138-1*** |
| hsa-miR-4507 | **hsa-miR-373*** |  | **bta-miR-409b** |
| **bta-miR-2486** | **hsa-miR-658** |  | **bta-miR-2487** |
| **mmu-miR-3092*** | **hsa-miR-491-5p** |  | **mmu-miR-673-3p** |
| **mmu-miR-3104-5p** | **hsa-miR-4751** |  | **mmu-miR-3110*** |
|  |  |  | **mmu-miR-128-1*** |
|  |  |  | **mmu-miR-1946a** |
|  |  |  | **mmu-miR-138-1*** |
|  |  |  | **hsa-miR-4787-5p** |
|  |  |  | **hsa-miR-4783-3p** |
|  |  |  | **hsa-miR-4469** |

Note: The putative miRNA binding sites were predicted using Microinspector online software based on the human, mouse, cow and sheep miRNA databases. By default, the hybridization temperature is 37°C, and free energy cut-off -20 kcal/mol. There were 14, 4, 4 and 9 putative miRNA binding sites disrupted by the four SNPs, which are presented in normal font, and there were 3, 13, 2 and 14 putative miRNA binding sites created by the four SNPs, which are presented in bold font. MiR-3957-5p and miR-188 were selected for further study.
